# 262. COVID-19 associated fungal co-infections in Solid Organ Transplant Recipients: A single center case series

**DOI:** 10.1093/ofid/ofac492.340

**Published:** 2022-12-15

**Authors:** Akshay M Khatri, Jacques Simkins, Neeraj Sinha, Anita Phancao, Gaetano Ciancio, Lilian M Abbo, Giselle Guerra, Yoichiro Natori, Shweta Anjan

**Affiliations:** UnityPoint Health, Des Moines, Iowa; University of Miami Miller School of Medicine and Miami Transplant Institute, Jackson Health System, Miami, Florida; Miami Transplant Institute, Jackson Health System, Miami, Florida; Miami Transplant Institute, Jackson Health System, Miami, Florida; Miami Transplant Institute, Jackson Health System, Miami, Florida; University of Miami Miller School of Medicine and Miami Transplant Institute, Jackson Health System, Miami, Florida; Miami Transplant Institute, Jackson Health System, Miami, Florida; University of Miami Miller School of Medicine and Miami Transplant Institute, Jackson Health System, Miami, Florida; University of Miami Miller School of Medicine and Miami Transplant Institute, Jackson Health System, Miami, Florida

## Abstract

**Background:**

During the ongoing Coronavirus disease of 2019 (COVID-19) pandemic, there have been increasing reports of viral, bacterial and fungal co-infections. Two COVID-19-associated fungal infections (CFIs) have been identified – COVID-19 associated pulmonary aspergillosis (CAPA) and COVID-19 associated mucormycosis (CAM), but incidence and occurrence in solid organ transplant recipients (SOTRs) is limited. We describe our experience with CFIs in SOTRs with COVID-19.

**Methods:**

In a single center retrospective study at a large volume transplant center in South Florida, USA, we included adult SOTRs (≥18 years) diagnosed with COVID-19 between March 1^st^ 2020 and January 31^st^ 2022, with subsequent diagnosis of CFI. We collected information related to demographics, comorbidities, COVID-19 diagnosis and therapeutics, and CFI diagnostics and management. Data obtained was analyzed descriptively.

**Results:**

We identified 612 SOTRs with COVID-19, of which 23 (3.8%) were diagnosed with CFIs.

The patients were predominantly male (17/23, 73.9%), with median age of 59 years (range 43-79) [**Table 1**]. Twenty (86.9%) were kidney transplant recipients. Majority of SOTRs had lymphopenia (18/23, 78.3%) with elevated inflammatory markers at time of COVID-19 diagnosis. They received most commonly remdesivir and corticosteroids for COVID-19, with 22 (95.6%) needing intensive care unit admission and 19 (82.6%) needing continuous renal replacement therapy.

CFIs were diagnosed at median 21 days (range, 3-161) after initial COVID-19 diagnosis. Probable CAPA was diagnosed in most patients (16/23, 69.6%), with CAM noted in 1 patient [**Table 2**]. 34.8% (8/23) had specific fungal species identified, with elevated fungal markers noted in 95.6% (22/23). Concurrent or prior cytomegalovirus DNAemia was noted in 26.1% (6/23). Patients were followed for median 70 days (range, 19-572), with median hospitalization duration 56 days (range, 7-204). Mortality was noted in 73.9% (17/23).

**Table 1:**
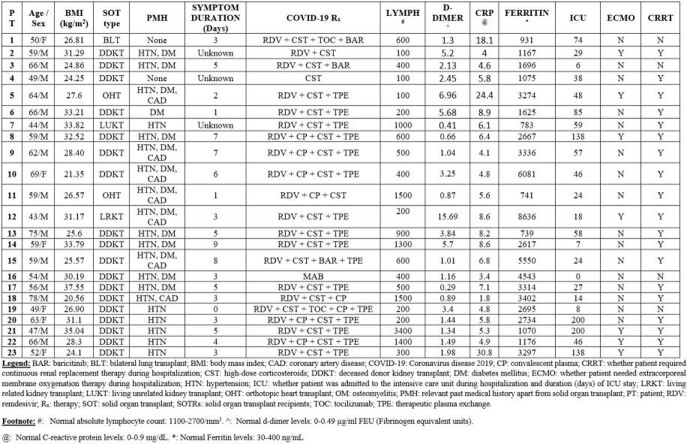
COVID-19 related clinical characteristics in study patients (N=23)

**Table 2:**
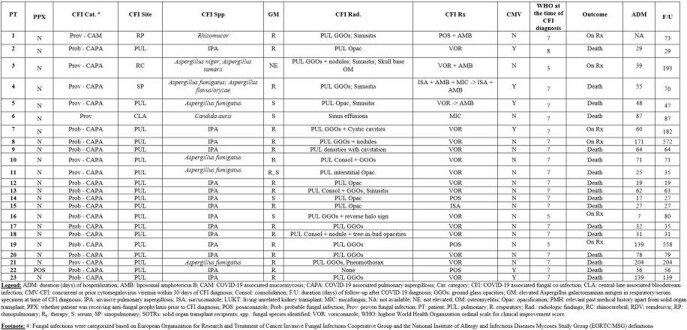
CFI-related clinical characteristics in study patients (N=23)

**Conclusion:**

Fungal co-infections were noted in a small proportion of our SOTRs, with poor outcomes. Transplant physicians should have a high suspicion for early diagnosis and treatment of CFI. Further studies are needed to determine predictors for CFI and role for anti-fungal prophylaxis.

**Disclosures:**

**All Authors**: No reported disclosures.

